# Evaluation of reference genes for real-time RT-PCR expression studies in the plant pathogen *Pectobacterium atrosepticum*

**DOI:** 10.1186/1471-2229-7-50

**Published:** 2007-09-21

**Authors:** Gunnhild W Takle, Ian K Toth, May B Brurberg

**Affiliations:** 1 Norwegian Institute for Agricultural and Environmental Research, Plant Health and Plant Protection Division, Høgskoleveien 7, 1432 Ås, Norway; 2 Norwegian University of Life Sciences, Institute for Chemistry, Biotechnology and Food Science, PO Box 5003, 1432 Ås, Norway; 3 SCRI, Invergowrie, Dundee DD2 5DA, UK

## Abstract

**Background:**

Real-time RT-PCR has become a powerful technique to monitor low-abundance mRNA expression and is a useful tool when examining bacterial gene expression inside infected host tissues. However, correct evaluation of data requires accurate and reliable normalisation against internal standards. Thus, the identification of reference genes whose expression does not change during the course of the experiment is of paramount importance. Here, we present a study where manipulation of cultural growth conditions and *in planta *experiments have been used to validate the expression stability of reference gene candidates for the plant pathogen *Pectobacterium atrosepticum*, belonging to the family *Enterobacteriaceae*.

**Results:**

Of twelve reference gene candidates tested, four proved to be stably expressed both in six different cultural growth conditions and *in planta*. Two of these genes (*recA *and *ffh*), encoding recombinase A and signal recognition particle protein, respectively, proved to be the most stable set of reference genes under the experimental conditions used. In addition, genes *proC *and *gyrA*, encoding pyrroline-5-carboxylate reductase and DNA gyrase, respectively, also displayed relatively stable mRNA expression levels.

**Conclusion:**

Based on these results, we suggest *recA *and *ffh *as suitable candidates for accurate normalisation of real-time RT-PCR data for experiments investigating the plant pathogen *P. atrosepticum *and potentially other related pathogens.

## Background

Real-time reverse transcription polymerase chain reaction (real-time RT-PCR) has become the preferred method for studying low-abundant mRNA expression [1]. The high sensitivity and specificity of RT-PCR makes it a particularly useful and powerful technique for monitoring the mRNA expression of pathogen genes during host infection, where the pathogen's expression profile is often masked by the much higher concentration of host RNA. However, the study of pathogen gene expression inside infected host tissue poses some problems, as there is no straightforward way of measuring the total pathogen RNA concentration. An increase in target transcript at different time points after infection could either come from an up-regulation of transcription or merely from an increase in the pathogen population inside the host tissue, or both. Therefore, normalisation of the data against reference genes (i.e., genes whose expression do not change under the various experimental conditions) is an important step in the quantification of gene expression. Reference genes are also used to correct for differences between samples, such as variation in the total quantity of RNA and variation in RT-PCR efficiency. Therefore, it is of paramount importance to find stably expressed reference genes, as the reliability of the normalised expression data is only as good as the reliability of the reference gene(s). Any variation in reference gene expression could in principle mask real positives as well as create false positives [2-5]. To obtain a reliable normalisation, the use of more than one reference gene is recommended [6-8]. The expression of typical prokaryotic housekeeping genes has been reported to be highly variable under most experimental conditions [9].

*Pectobacterium atrosepticum *(formerly known as *Erwinia carotovora *subspecies *atroseptica *[10,11]) is an important bacterial pathogen of potato in temperate regions, where it causes blackleg of plants and soft rot of tubers by the utilization of a huge machinery of plant cell wall degrading exoenzymes mainly encompassing pectinases, cellulases and proteases. The bacterium lies dormant in the plant or tuber until conditions are favourable for infection [12-14]. The recently published genome of *P. atrosepticum *strain SCRI1043 has provided valuable tools for examining different aspects of pathogenesis [15]. Our own (unpublished) initial RT-PCR-based studies on differential gene expression in *P. atrosepticum *during potato infection revealed problems related to reliability of reference genes. Therefore, we have conducted a wider search for reference gene candidates, with the aim of producing a set of reference genes that can be applied to future real-time RT-PCR experiments with *P. atrosepticum*, and potentially other bacteria, in infected plant material.

We have used a combination of different growth media, temperatures and growth phases to test the expression stability of twelve reference gene candidates in *P. atrosepticum*, and completed the test with an experiment in infected potato leaves. The following reference gene candidates were included in this study: Signal recognition particle protein (*ffh*), glutamine synthetase (*glnA*), DNA gyrase (*gyrA*), pyrroline-5-carboxylate reductase (*proC*), recombinase A (*recA*), transcription termination factor Rho (*rho*), 50S ribosomal protein L9 (*rplI*), 50S ribosomal subunit protein L17 (*rplQ*), DNA topoisomerase I (*topA*), nucleoside-specific channel-forming protein (*tsx*), maltose-binding periplasmic protein (*malE*) and 16S ribosomal RNA (*16S*).

## Results

### Selection of twelve reference gene candidates for real-time RT-PCR

On the basis of the *P. atrosepticum *SCRI1043 genome sequence [15], a set of twelve reference gene candidates were selected for an initial real-time RT-PCR study. The genes were selected from different parts of the genome to minimize the chance of transcriptional coupling affecting the results. They were also selected to encode proteins involved in different metabolic activity except for the ribosomal genes, in order to minimize the chances of a global co-regulation. The ribosomal gene 16S rRNA is a commonly used reference gene in many real-time RT-PCR experiments, including studies on former *Erwinia *species [16-23]. *GlnA *has been used as a reference gene in *Streptococcus pneumoniae *real-time experiments [24] and *gyrA *has been used in studies on different bacteria including a very recent study on the plant pathogen *Pseudomonas syringae *pv. *tomato *[21,25,26]. *ProC *and *rho *have been tested as reference genes in similar studies of other bacteria, although no plant pathogens to our knowledge [27,28]. The genes *rplI *and *rplQ *are part of a group of ribosomal proteins that have also recently been used as reference genes in real-time PCR experiments on both prokaryotes and eukaryotes [29-32]. *RecA *is a common housekeeping gene that has been used as reference gene in studies on various bacteria (e.g. [20,27,33-35]) and was recently found to be a good reference gene for certain studies of *P. atrosepticum *(I. K. Toth, unpublished results). The genes *ffh*, *topA*, *tsx *and *malE *have, to our knowledge, not been reported as reference genes previously. The latter four genes were selected merely on the basis of either being well-known housekeeping genes in other bacteria (*ffh, topA*), or known to be expressed during different growth conditions in *P. atrosepticum *(*tsx, malE*) [36].

### Expression analysis of reference gene candidates in cultures

**Figure 1:**
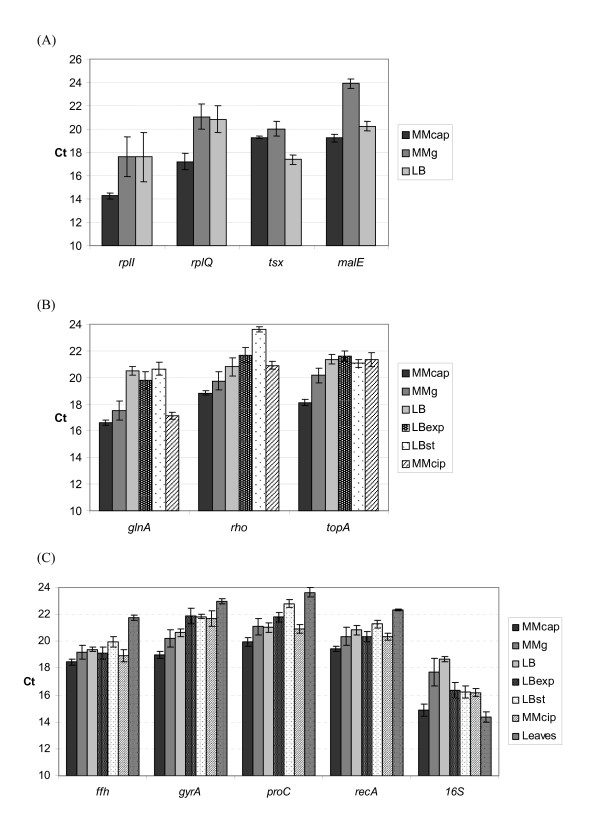
**Expression levels of reference gene candidates during different cultural conditions**. Gene expression levels (represented by absolute Ct values) of (A) *rplI, rplQ, tsx *and *malE *in MMcap, MMg and LB, (B) *glnA, rho *and *topA *in MMcap, MMg, LB, LBexp, LBst and MMcip and (C) *ffh, gyrA, proC, recA *and *16S *in MMcap, MMg, LB, LBexp, LBst, MMcip and infiltrated leaflets. For explanations of abbreviations, see Table 3. Each bar represents the mean of three samples from independent RNA isolations from three cultures, except for the leaves bar, which come from two independent RNA isolations from a pool of three vacuum infiltrated leaflets. Error bars indicate standard deviations.

An initial study of expression of all the genes selected as reference gene candidates (*ffh, glnA, gyrA, proC, recA, rho, rplI, rplQ, topA, tsx, malE, 16S*) was performed in three different growth media (MMcap, MMg and LB) at 15°C. The genes *rplI, rplQ, tsx *and *malE *showed large variations in Ct values in the different media (Fig. 1A), and were therefore discarded from further studies. The eight remaining reference gene candidates (*ffh, glnA, gyrA, proC, recA, rho, topA, 16S*) were subjected to a second experiment testing the effect of increased temperature (27°C), different pectin (citrus) and growth phase (exponential vs. stationary) on expression. The genes *glnA, rho*, *topA *and *16S *were expressed at varying levels while the expression of the reference gene candidates *ffh, gyrA, proC *and *recA *was relatively stable under all culture conditions (Fig. 1BC). To get a statistical evaluation of the results, we analysed the Ct values by the Excel-based program BestKeeper [7,37]. The descriptive statistics of the eight genes based on expression in six different culture conditions are given in Table 1. BestKeeper expresses the Ct range of each individual gene as the extreme values of Ct towards the geometric mean Ct, and gives their standard deviations, hence providing an evaluation of the expression stability of each reference gene candidate (Table 1, (min, max) [x-fold] and SD [± x-fold], respectively). Based on this analysis, the *ffh *gene was ranked as most stably expressed and *recA *as the second most stably expressed gene, across all growth conditions. Due to their high standard deviations (Table 1, SD [± x-fold]), as well as their overall variable expression across all cultural growth conditions (Fig. 1B), the candidates *glnA, rho *and *topA *were discarded from subsequent tests. We decided to include the *16S *candidate, despite the fact that it had a fairly large Ct range and a high standard deviation, because of its common use as a reference gene (e.g. [16,17,21-23]).

**Table 1:** Descriptive statistics of reference gene expression across all cultural growth conditions by BestKeeper^1^

Gene	GM [Ct]	(min, max) [Ct]	SD [± Ct]^2^	(min, max) [x-fold]^3^	SD [± x-fold]^2^
*ffh*	19.17	18.23, 20.34	0.48	-1.92, 2.25	1.39
*glnA*	18.62	16.37, 21.14	1.62	-3.22, 3.70	3.07
*gyrA*	20.85	18.72, 22.56	0.94	-3.87, 2.98	1.92
*proC*	21.25	19.66, 23.16	0.73	-2.28, 2.69	1.66
*recA*	20.42	19.21, 21.46	0.55	-2.16, 1.96	1.47
*rho*	20.88	18.66, 23.84	1.24	-3.65, 5.62	2.35
*topA*	20.57	17.92, 22.03	0.99	-5.56, 2.56	1.98
*16S*	16.62	14.49, 18.89	1.04	-2.83, 3.03	2.06

^1^Values calculated from 18 samples (6 different culture conditions, each with 3 biological replicates). ^2^Based on the inspection of the standard deviations (SD), genes can be ranked from the most stably expressed (*ffh*), exhibiting the lowest SD, to the least stably expressed (*glnA*), exhibiting the highest SD. Genes with SD [± Ct] > 1 are considered to be inconsistent by BestKeeper. ^3^The (min, max) [x-fold] values are the x-fold under- or over-expression of samples towards the GM [Ct] according to the equations *min [x-fold] = E*^*min [Ct]-GM [Ct] *^and *max [x-fold] = E*^*max [Ct]-GM [Ct]*^, respectively, where E is the specific PCR efficiency.

GM [Ct], geometric mean of Ct; (min, max) [Ct], the extreme values of Ct; SD [± Ct], standard deviation of Ct; (min, max) [x-fold], extreme values of expression levels expressed as an absolute x-fold over- or under-regulation coefficient where min [x-fold] = first value and max [x-fold] = last value; SD [± x-fold], standard deviation of the absolute regulation coefficients [7].

### Expression of selected reference gene candidates in infected plant material

**Figure 2:**
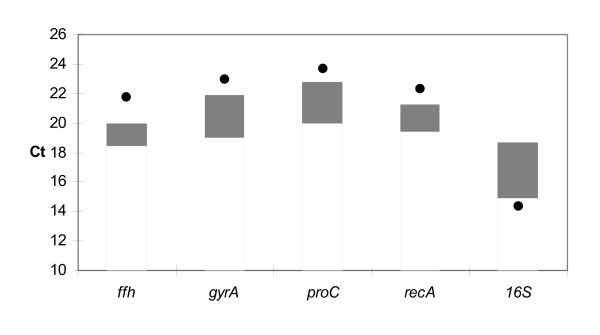
**Overall expression stability of *ffh, gyrA, proC, recA *and *16S *in different culture conditions and infiltrated leaflets**. Overall stability across different growth conditions for the five genes *ffh, gyrA, proC, recA *and *16S*. Grey bars represent range of Ct values across all cultural growth conditions, while dots represent Ct values for experiments on infiltrated leaflets. For explanations of abbreviations, see Table 3.

From the BestKeeper analysis, the four most stably expressed reference gene candidates, *ffh, recA, proC *and *gyrA*, as well as the *16S *gene, were examined for expression after infiltration of detached potato leaflets with *P. atrosepticum*. In the experiment, leaflets infiltrated with bacteria showed clear rotting symptoms after 21 hours, while control leaflets infiltrated with only sterile buffer appeared fresh and healthy. The expression in culture and *in planta *cannot be directly compared, as the amount of starting concentration of bacteria or bacterial RNA cannot be equalised, but the expression can be compared with respect to variation between different genes. RT-PCR analysis of RNA collected after 21 hours incubation showed that four of the candidate reference genes had similar expression levels in all samples, whereas the *16S *gene showed a clear up-regulation of mRNA expression (lower Ct value) in infected plant material compared to in culture, and relative to the other reference gene candidates (Fig. 1C and Fig. 2). The *16S *primers also gave a weak signal in the non-infected leaflets (results not shown). None of the other primer pairs gave amplicons in the non-infected leaflets, confirming the absence of *P. atrosepticum *in these controls. To get a statistical evaluation of the candidates, we performed another statistical test to rank them according to expression stability.

### Statistical analysis of real-time RT-PCR data by geNorm

A statistical analysis of the expression data from the five candidate reference genes, which were subjected to all experimental growth conditions, was performed using geNorm [6,38]. This program determines the pairwise variation of a reference gene with all other reference genes and defines the reference gene-stability measure M as the average pairwise variation of a particular gene with all other reference genes. Genes with the lowest M values have the most stable expression. Stepwise exclusion of the gene with the highest M value results in a combination of two constitutively expressed reference genes that have the most stable expression in the tested samples. From this analysis, the genes *ffh *and *recA *were estimated to have the lowest M values and hence the highest stability, while *16S *gave the highest M value (lowest stability) (Table 2). geNorm also calculates the pairwise variation (V_n/n+1_) between the normalisation factors to determine the optimal number of reference genes needed for normalisation. The cut-off threshold was set to V = 0.15, below which the inclusion of another reference gene is not required, as suggested by Vandesompele *et al*. [6]. A low (V_n/n+1_) value shows that the inclusion of the (n+1)th gene has no significant effect. As shown by a V value of 0.118 (Table 2), the use of the two most stably expressed genes, *ffh *and *recA*, as reference genes are sufficient for a reliable data normalisation in this expression analysis in *P. atrosepticum*.

**Table 2:** Ranking of reference genes by geNorm

Genes	Average expression stability M	Pairwise variations V
*ffh*	0.334	-
*recA*	0.334	0.118
*proC*	0.371	0.166
*gyrA*	0.533	0.288
*16S*	0.909	-

Ranking of the most stably expressed genes from top to bottom and determination of the optimal number of genes needed for normalisation. The average expression stability value M was obtained by calculating the M for each gene and excluding the gene with the highest M (lowest stability) for the next calculation round. The optimal number of reference genes needed for normalisation is determined by the pairwise variation (V) between the normalisation factors. The cut-off value of V was set to 0.15, below which the inclusion of another reference gene has no significant effect, as suggested by Vandesompele *et al*. [6].

## Discussion

Real-time RT-PCR is becoming an important technology for studying host-pathogen interactions. However, proper and highly reliable reference genes are needed for normalisation of data, as normalisation by total pathogen RNA in mixed host-pathogen samples is usually not possible. Here, we describe a set of reference genes that can be used to normalise gene expression in the potato pathogen *Pectobacterium atrosepticum *and potentially other related pathogens. We identified several genes that showed only minor variations in expression under a range of growth conditions. The expression of these genes was then further analysed using the Excel-based programs BestKeeper [7,37] and geNorm [6,38]. Both programs have been used in several recent studies [39-44]. While geNorm requires that the data be converted to relative expression values, BestKeeper allows for the input of Ct values.

From the statistical analyses it was concluded that two genes, *recA *and *ffh *encoding recombinase A and signal recognition particle (SRP) protein, respectively, were particularly stably expressed. Also *proC *and *gyrA *were relatively stably expressed under the conditions used in this study. On the other hand, the *16S *gene was not stably expressed in the different culture conditions used in this study. This gene was included in our analyses because of its extensive use as a reference gene in several real-time PCR studies [16-23]. Although commonly used, some reports suggest that this gene is also under regulatory control [9,45]. In addition, we discovered that *16S *was amplified from leaf material that was not inoculated with the pathogen. Since *16S *genes are very conserved in different bacterial species and even within eukaryotic chloroplasts and mitochondria, this "non-specific" amplification could be from plant material in the samples as well as from bacteria naturally present in the phyllosphere. This result suggests that *16S *is inappropriate as a reference gene when not analysing pure cultures, such as complex host-pathogen samples. Another disadvantage of normalising against *16S *is that the cellular quantity of ribosomal RNA is much higher than that of mRNA. This makes it necessary to dilute the cDNA samples prior to real-time analysis, thus risking dilution errors. Also, while mRNAs have a rapid turn-over according to the bacteria's needs, the rRNA is only degraded under certain stress conditions or when the molecule is defective [46], hence, the rRNA population is not comparable to the mRNA population.

The choice of an acceptable level of reference gene expression variability depends on the degree of sensitivity that is demanded for each experiment. Obviously, the goal is always to strive towards finding reference genes with the lowest possible expression variability. However, even a reference gene expressing some variability over the course of the experiment may be sufficient to detect target gene expression variations as long as these are larger than for the reference gene. Generally, reference genes with geNorm expression stability measures below 1.0 have been regarded as suitable for normalisation in some studies [28,39,44] and are also below the geNorm default limit of M = 1.5 [6]. In this study, the five reference genes *ffh*, *recA*, *proC*, *gyrA *and *16S *all have an expression stability measure below 1.

Analyses by geNorm suggest that the combination of *ffh *and *recA *is the optimal set of reference genes for studying differential gene expression in *P. atrosepticum *by real-time RT-PCR under the various conditions applied in this study. These conditions included two different growth temperatures, exponential and stationary growth phase, rich and minimal growth medium with two different types of pectin, as well as infiltrated potato leaves. However, it has been recommended that at least three reference genes should be used for correct normalisation of real-time RT-PCR data [6]. The results from this study suggest that *proC *could be used together with *ffh *and *recA*. However, increasing the number of reference genes means increasing the workload and cost. In addition, applying a large reference gene set could pose problems with limited sample availability. The use of a single reference gene is generally acceptable, but this gene should be subjected to extensive studies before use to ensure its stability [8].

All methods for RNA detection face problems concerning stability of RNA as well as sensitivity and specificity of detection [8,47]. Studying pathogens inside host tissues poses a further complication, as the amount of pathogen RNA often becomes vanishingly small compared to host RNA. Although there are a few exceptions (e.g. [48-50]) it is not currently possible to avoid having to deal with mixed eukaryote – prokaryote RNA when looking at pathogens inside host tissues. It is thus of utmost importance to find good reference genes for normalisation of real-time RT-PCR data from mixed host – pathogen RNA samples. Bacterial gene expression is highly diverse, and there is unlikely to be a single universally and stably expressed prokaryotic housekeeping gene. Therefore, we support the general notion that tests should be conducted in any real-time RT-PCR experiment before deciding which genes to use as reference genes for a particular study, and the recommendations of using more than one reference gene, in particular when studying pathogen expression inside infected host tissue [6,8,9,28,39,51,52].

## Conclusion

Here we present a study where manipulations of growth conditions for *P. atrosepticum *were used in order to find a set of reliable reference genes for monitoring bacterial gene expression inside infected plant tissue. A set of two reference genes, *ffh *and *recA*, proved to be the optimal set for use under the conditions applied. To our knowledge, this is the first time reference genes for studying gene expression by real-time PCR have been systematically examined in a plant pathogenic bacterium. The evaluated set of genes in this study could also provide valuable guidelines for reference gene selection when working on mRNA expression in other bacterial pathogens, in particular from the family *Enterobacteriaceae*.

## Methods

### Culture conditions and bacterial infiltration of potato leaves

*Pectobacterium atrosepticum *strain SCRI1043 was obtained from the SCRI bacterial collection. The bacteria were grown in a variety of media at 15°C or 27°C as described in Table 3. Pectin, polygalacturonic acid and arabinogalactan were added to two of the growth media in order to trigger different responses in *P. atrosepticum *gene transcription. Two different types of pectin, citrus pectin (cip) and cabbage pectin (cap) were used, as this could in principle affect the transcriptional response of the bacterium. The temperature of 15°C was selected based on optimal expression of key enzymes involved in the breakdown of the plant cell walls (e.g. pectate and pectin lyase). The temperature of 27°C was selected as the optimum growth temperature for *P. atrosepticum *[53]. In addition, gene expression levels at different growth phases were tested, as these may vary during plant infection. Thus, bacteria were sampled from both exponential and stationary phase in LB medium (Table 3). For leaf infiltration, overnight LB-cultures of *P. atrosepticum *grown at 27°C were pelleted and resuspended in 10 mM MgSO_4_. Leaflets from potato cv. Bintje were vacuum infiltrated with a suspension of 10^7 ^bacterial cells/ml for ~15 minutes under low vacuum using a water pump. Negative control leaflets were infiltrated with 10 mM MgSO_4 _without bacteria. After infiltration, leaflets were placed on moist filter paper in Petridishes (3–5 leaflets per dish), and incubated at 18°C. This temperature was selected to mimic conditions at which *P. atrosepticum *optimally causes blackleg and soft rotting symptoms [13]. Samples were harvested 21 hours after infiltration, at which point the leaves showed clear rotting symptoms, flash-frozen in liquid nitrogen and kept at -80°C until RNA extraction.

**Table 3:** *P. atrosepticum *cultural growth conditions

Name	Medium	Temp (°C)	Phase (A_600_)
MMcap	M9 mm, cap (0.05 %), pga (0.125 %), abg (0.05 %)	15	0.4
MMg	M9 mm, glucose (0.2 %)	15	0.4
LB	LB	15	0.4
LBexp	LB	27	0.4
LBst	LB	27	2.0
MMcip	M9 mm, cip (0.5 %), pga (0.5 %), abg (0.5 %)	27	0.4

M9 mm, M9 minimal medium; LB, Luria Bertani broth; cap, cabbage pectin; pga, polygalacturonic acid; abg, arabinogalactan; cip, citrus pectin. M9 minimal medium and LB medium was made according to Sambrook *et al*. (1989) [56]. All cultural conditions included shaking at 200 rpm.

### RNA isolation from cultures and infected plant material

For bacterial cultures, total RNA was isolated from ~1 × 10^9 ^cells using the RNeasy mini kit (QIAGEN). On-column DNase digestion using the RNase-Free DNase Set (QIAGEN) was included in the protocol. To remove DNA, it was necessary to include an additional DNAse treatment using RQ_1 _RNase-Free DNase (Promega). This was followed by phenol:chloroform:isoamyl alcohol extraction (25:24:1) and precipitation with ethanol. The RNA pellet was dissolved in DEPC-treated water. Total RNA from leaf material was isolated using TRIzol Reagent (Invitrogen). The RNA was then subjected to a phenol:chloroform:isoamyl alcohol extraction to increase the purity, after which the samples were subjected to two subsequent DNase treatments with RQ1 RNase-Free DNase (Promega), followed by phenol:chloroform:isoamyl alcohol extractions and precipitation with ethanol. Mixed plant-bacterial total RNA was treated with MICROB *Enrich *(Ambion), according to the manufacturer's recommendations. Briefly, mixed host-pathogen total RNA samples were incubated together with oligonucleotides that capture eukaryotic polyadenylated mRNA as well as 28S and 18S rRNA. The oligonucleotide-hybridized mRNA and rRNA were then removed using magnetic beads. The enriched bacterial RNA was precipitated and resuspended in RNase-free water. All the RNA samples were assessed for quality by agarose gel electrophoresis, and quantified using a GeneQuant spectrophotometer (GE Healthcare). Absence of genomic DNA contamination was confirmed by PCR.

### Reverse transcription, real-time PCR and data analysis

Reverse transcription was performed on 1 μg RNA using SuperScript III Reverse Transcriptase (Invitrogen). Primers for real-time PCR, listed in Table 4, were designed using the program PrimerExpress (Applied Biosystems) based on the *P. atrosepticum *SCRI1043 genome sequence [15,54]. Real-time PCR reaction mixtures contained 12.5 μl 2×SYBRGreen PCR MasterMix (Applied Biosystems), 10 pmoles of each primer, 2 μl template (10× diluted cDNA from cultures, 3× diluted cDNA from leaf samples), and sterile distilled water to a total volume of 25 μl. Because of the high abundance of 16S rRNA, cDNA was diluted 100 fold more when analysing *16S *expression. Thermal conditions were 95°C for 10 minutes followed by 40 cycles of 95°C for 15 seconds and 60°C for 1 minute. For detection of primer dimerisation or other artifacts of amplification, a melting-curve analysis was performed immediately after completion of the real-time PCR (95°C for 15 seconds, 60°C for 15 seconds, and then slowly increasing the temperature to 95°C at a 2 % ramp rate, with continuous measurement of fluorescence). All reactions were performed in triplicate. Three non-template controls were included for each primer pair. Quantification of gene expression was performed using a 7900 HT Real-Time PCR System (Applied Biosystems) and real-time data were analysed using the ABI PRISM 7900 HT Software Tool (Applied Biosystems). The amplification efficiency (E) for all primers was determined using a cDNA pool dilution series from all culture conditions and the Relative Expression Software Tool (REST) for calculations [55]. These efficiencies were included in all subsequent analyses. Results were evaluated using BestKeeper [7,37] and geNorm [6,38].

**Table 4:** Primers used in this study

Gene	Forward primer (5' – 3')	Reverse primer (5' – 3')	Amplicon
*ffh*	ATGGGCGATGTGCTTTCACT	TCAAACCCATCGCCTTTCTT	101
*glnA*	TCCAGCAGCTAACCCGTACC	GGTTTTTGTCCATCGCATCG	101
*gyrA*	CTGCCGTGAGTGAGTACCCA	AACCTGAACCGCACCAACC	101
*proC*	CACAGCTGATGCAGAGCGTC	GAAGAAATAGGCCGGTGCG	101
*recA*	GGTGAGCTGGTTGATCTGGG	GCATTCGCTTTACCCTGACC	101
*rho*	TGACTGTGTGCTGATGGTGCT	CGTCAAACGTTGACGCAATG	101
*rplI*	ACCATCGCGTCTAAAGCAGG	TTAGCAATGTCAACACCGGC	101
*rplQ*	CAAGACCGACAGCGTTGCTA	CGGGCCCAGTTCATTAAACA	91
*topA*	TGCGTATTTCGTATTGCGTGA	TCTTCTACCAGCGGTGCCC	96
*tsx*	CTCTCTGATGGGCGGTAACG	TGGAGTTGTTAGTGCGGCTTG	101
*malE*	TCCGCTGATTAAGGATGACGA	GCGTCCAAACTTCCTGCATT	101
*16S*	CAATATTCCCCACTGCTGCC	CACCTAGGCGACGATCCCT	101

## Competing interests

The author declares that there are no competing interests.

## Authors' contributions

GWT planned and performed the experiments and analyses and prepared the first draft of the manuscript. MBB participated in planning of experiments, general supervision and manuscript preparation. IKT provided the *P. atrosepticum *SCRI1043 strain and the basis for selecting the *recA *gene, as well as critical reading of the manuscript. All authors have read and approved the final manuscript.
